# Differences in the force system delivered by different beta-titanium wires in elaborate designs

**DOI:** 10.1590/2177-6709.20.6.089-096.oar

**Published:** 2015

**Authors:** Renato Parsekian Martins, Sergei Godeiro Fernandes Rabelo Caldas, Alexandre Antonio Ribeiro, Luís Geraldo Vaz, Roberto Hideo Shimizu, Lídia Parsekian Martins

**Affiliations:** 1 Adjunct professor, Universidade Estadual Paulista Julio de Mesquita Filho (UNESP), School of Dentistry, Department of Orthodontics, Araraquara, São Paulo, Brazil.; 2 Adjunct Professor, Universidade Federal do Rio Grande do Norte (UFRN), Department of Dentistry, Natal, Rio Grande do Norte, Brazil.; 3 Professor, Universidade Potiguar (UnP) and Associação Brasileira de Odontologia (ABO), Specialization course in Orthodontics, Natal, Rio Grande do Norte, Brazil.; 4 Professor, Universidade Estadual Paulista Julio de Mesquita Filho (UNESP), School of Dentistry, Department of Prosthesis and Dental Material, Araraquara, São Paulo, Brazil.; 5 Professor, Universidade Tuiuti do Paraná (UTP), School of Dentistry, Curitiba, Paraná, Brazil.; 6 Professor, Universidade Estadual Paulista Julio de Mesquita Filho (UNESP), School of Dentistry, Department of Orthodontics and Pediatric Dentistry, Araraquara, São Paulo, Brazil.

**Keywords:** Orthodontics, Tooth movement, Orthodontic wires

## Abstract

**Objective:**

Evaluation of the force system produced by four brands of b-Ti wires bent into an elaborate design.

**Methods:**

A total of 40 T-loop springs (TLS) hand-bent from 0.017 x 0.025-in b-Ti were randomly divided into four groups according to wire brand: TMA^TM^(G1), BETA FLEXY^TM^ (G2), BETA III WIRE^TM^ (G3) and BETA CNA^TM^ (G4). Forces and moments were recorded by a moment transducer, coupled to a digital extensometer indicator adapted to a testing machine, every 0.5 mm of deactivation from 5 mm of the initial activation. The moment-to-force (MF) ratio, the overlapping of the vertical extensions of the TLSs and the load-deflection (LD) ratio were also calculated. To complement the results, the Young's module (YM) of each wire was determined by the slope of the load-deflection graph of a tensile test. The surface chemical composition was also evaluated by an energy dispersive X-ray fluorescence spectrometer.

**Results:**

All groups, except for G2, produced similar force levels initially. G3 produced the highest LD rates and G1 and G4 had similar amounts of overlap of the vertical extensions of the TLSs in "neutral position". G1 and G3 delivered the highest levels of moments, and G2 and G3 produced the highest MF ratios. b-Ti wires from G3 produced the highest YM and all groups showed similar composition, except for G2.

**Conclusion:**

The four beta-titanium wires analyzed produced different force systems when used in a more elaborate design due to the fact that each wire responds differently to bends.

## Resumo

**Objetivo:**

avaliar os sistemas de força produzidos por quatro marcas de fios de -Ti, dobrados em formas complexas.

**Métodos:**

quarenta molas T-Loop (TLS) dobradas à mão, confeccionadas a partir de fios de -Ti, calibre 0,017" x 0,025", foram aleatoriamente divididas em quatro grupos, de acordo com a marca do fio: TMA (G1), BETA FLEXY (G2), BETA III Wire (G3) e BETA CNA (G4). Forças e momentos foram registrados por um transdutor de momento - acoplado a um extensômetro digital, adaptado a uma máquina de ensaios -, a cada 0,5mm de desativação, a partir de 5mm da ativação inicial. A proporção momento-força (M/F), a sobreposição das extensões verticais das TLSs e a relação carga-deflexão (C/D) também foram calculadas. Para completar os resultados, o módulo de Young (MY) de cada fio foi determinado pelo declive no gráfico de carga-deflexão em um ensaio de tração. Ademais, a composição química da superfície foi avaliada por um espectrômetro de fluorescência de raios X por energia dispersiva.

**Resultados:**

todos os grupos, exceto o G2, produziram níveis de força semelhantes, inicialmente. O G3 produziu as maiores taxas de C/D, enquanto G1 e G4 tiveram quantidades similares de sobreposição das extensões verticais das TLSs em posição neutra. G1 e G3 geraram os mais altos níveis de momentos, enquanto G2 e G3 produziram os mais altos índices de M/F. Os fios -Ti do G3 produziram o maior MY, e todos os grupos apresentaram composição semelhante, exceto G2.

**Conclusão:**

os quatro fios de beta-titânio analisados produziram diferentes sistemas de forças, quando utilizados para formas mais complexas, devido ao fato de que cada fio responde de forma diversa à confecção de dobras.

## INTRODUCTION

Beta-titanium (b-Ti) was introduced in Dentistry in the late 70's;^1^ since then it has been widely used in Orthodontics due to its excellent mechanical properties, such as high spring-back, low stiffness, high formability, and good weldability.^2^^-^ 6 After expiration of the patent^2^ on the first commercial brand of b-Ti (TMA^TM^, Ormco Co., Glendora, USA), the use of this alloy expanded drastically with a wide range of prices and quality. Even though there are several brands available to the clinician, only a few studies^2^^,^ 7 ^,^ 8 have been conducted in order to compare different b-Ti commercial brands. These studies, however, compare mechanical properties of b-Ti alloys either through tensile^2^^,^ 8 or through 3-point bending tests^7^ on straight pieces of wire. This might not represent the true behavior of the different b-Ti alloys when bends are placed in the wire or when more elaborate designs, such as loops, are used. 

It has been established that the T-loop spring (TLS) has the greatest ability to produce high moment-to-force (MF) ratios in order to control tooth movement when compared to other designs of springs. Several parameters of TLS have already been studied, such as spring's height,^9^^-^ 13 the location of the spring within inter bracket distance,^9^^,^ 12 ^-^ 15 the intensity and type of pre-activation,^11^^,^ 12 ^,^ 13 ^,^ 16 ^,^ 17 ^,^ 18 horizontal activation,^11^^,^ 13 alloy wire type,^3^^,^ 19 ^,^ 20 and stress relaxation;^21^ all of which can alter the MF ratio and force produced. However, differences between TLS manufactured with different b-Ti have not yet been systematically studied and are not completely understood. Thus, it is suggested that these alloys may present different biomechanical behavior, thereby affecting the force system released by the springs.

The objective of this study was to evaluate whether the behavior of four different brands of beta-titanium bent into an elaborate design (T-loop spring) are similar when forces, moments and MF ratios produced are compared. 

## MATERIAL AND METHODS

### Force system

**Figure 1:**
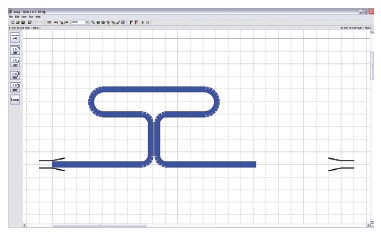
- Template developed in the Loop software (dHAL Orthodontic Software, Athens, Greece) used for the design of the TLS. The software allows the template to be printed in 1:1 ratio. Each square measures 1 mm^2^.

Sixty 6 x 10-mm T-loop springs (TLSs) were blindly bent out of four different commercial brands of 0.017 x 0.025-in b-Ti, using a Marcotte plier (Hu-Friedy dental instruments, Chicago, USA), a custom template (Fig 1). They were divided into four groups of 15 springs made of the same wire brand. The groups were previously labeled to assure impartiality of results. The wires used in the groups were TMA (Ormco Co., Glendora, USA) (G1), BETA FLEXY (Orthometric Imp.Exp.Ltda, Marília, Brazil) (G2), BETA III WIRE (Morelli Ortodontia, Sorocaba, Brazil) (G3) and BETA CNA (Ortho Organizers, INC., San Marcos, USA) (G4) (Table 1) TLSs were hand-bent in a random order; and out of the 15 TLSs bent, ten were randomly selected for testing. 

**Table 1:** - b-Ti wires used in the study.

**Group**	**Wire**	**Manufacturer**	**Batch**	**Measured dimensions (inches)**									
				**Height**	**Width**								
				**Mean**	**SD**		**Range**	***p* (95%)**	**Mean**	**SD**		**Range**	***p* (95%)**
1	TMA	Ormco Corporation	03F2F	0.0165	0.00	A	0.0	<0.001	0.024	0.00	B	0.0	0.007
2	BETA FLEXY	Orthometric Imp. Exp.	148	0.0161	0.00	B	0.0005		0.024	0.00	B	0.0	
3	BETA III TiMo	Morelli Ortodontia	1072448 100004	0.0165	0.00	A	0.0		0.0244	0.00	A	0.0005	
4	CNA	Ortho Organizers	401682D06	0.0165	0.00	A	0.0		0.0242	0.00	AB	0.0005	

**Figure 2:**
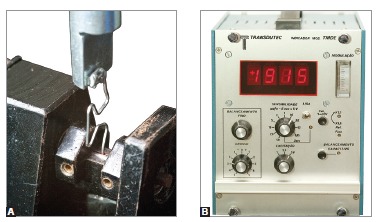
- A) Detail of the device used for the measurements: the bottom extremity of the TLS is firmly clamped by the moment transducer, while the top part of the TLS is tied to a bracket soldered to the universal testing machine, which records the horizontal force; B) Moment transducer used.

A universal testing machine (EMIC, São José dos Pinhais, Brazil), set up with a load cell of 0.1 kN, was coupled to a moment transducer and a digital extensometer indicator (Transdutec, São Paulo, Brazil) for the tests. The test speed was 5 mm/min and the digital extensometer excitation and sensitivity was 5 V and 0.5 mV/V, respectively (Fig 2).

**Figure 3:**
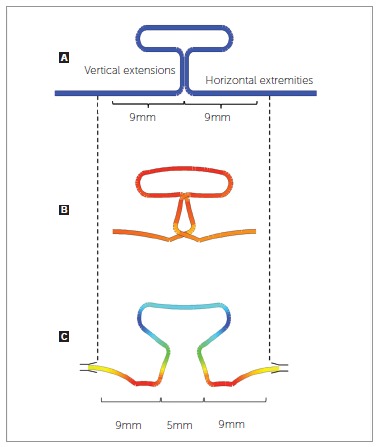
- A) Loop horizontal dimensions were marked to assure correct activation and TLS centralization; B) Neutral position of the TLS simulated by the Loop Software; C) TLS shape simulated by the Loop Software when positioned symmetrically in an IBD of 23 mm and activated 5 mm. Colored areas reflect stress distribution over the wire, going from red, being high stress areas; to dark blue, being low stress areas.

Prior to the test, concentrated bends were used to pre-activate the TLSs^22^ which were positioned symmetrically in an inter bracket distance (IBD) of 23 mm. At this distance, they were checked with a digital caliper and the testing device was zeroed. To assure the correct activation and the centralization of the TLSs, 9 mm were measured from the center of the loop towards each extremity of the horizontal extensions, and marked with a permanent marker (Fig 3). Those markings would allow the TLS to be correctly secured in place and centralized with the correct horizontal activation. The TLS was rigidly clamped to the test apparatus in one extremity and tied to a bracket on the other one with an elastomeric ligature.

**Figure 4:**
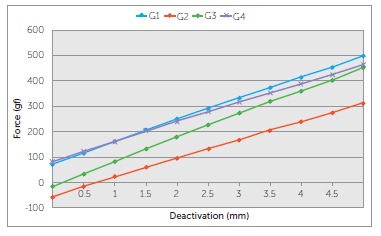
- Horizontal force (in grams-force) produced on deactivation by the four groups of b-Ti TLSs over a range of 5 mm.

After a horizontal activation of 5 mm, the horizontal force and moment developed were recorded for every 0.5 mm of deactivation at the extremity of the TLSs attached to the testing machine, and the MF ratios were calculated. Furthermore, the amount of overlap of the vertical extensions of the TLSs in neutral position (deformation assumed when the loop's extremities are placed parallel to the position that they will be once installed, producing only moments) was calculated by linear interpolation. The load-deflection (LD) ratio of each TLS was obtained by calculating the slope of the respective deactivation graph (Fig 4).

### Wire dimensions

The height and width of each wire were measured to the nearest 0.001 mm with a digital micrometer accurate to ± 1 µm (Mitutoyo, Kyoto, Japan). Five wires were taken from each group, totaling 20 readings, and the mean value was used in the subsequent calculations (Table 1).

### Mechanical properties

The sample comprised five 30-cm segments of each wire and was divided as presented in Table 1. The tensile test was performed on a universal testing machine (EMIC, São José dos Pinhais, Brazil) set up with a load cell of 5 kN and speed of 2 mm/min until rupture of the wire. Young's module (YM) was determined by the slope of the LD graph of the tensile test.^23^

### Chemical composition

An energy dispersive X-ray fluorescence spectrometer machine, model EDX-800, (Shimadzu Corporation, Kyoto, Japan), was used to determine the surface chemical composition of the wires in each group, using different wires from the same batch. Based on this analysis, it was assumed that the bulk composition was similar to surface compositions within the limits of accuracy. A fractographic image was obtained and the chemical composition was determined automatically in percentages.

### Statistical analysis

SPSS v.16.0 (SPSS Inc., Chicago, USA) statistical analysis software was used in this study. Kolmogorov-Smirnov test indicated normal distribution of data and one-way ANOVA test was used to identify differences among groups. Tukey post hoc test, at a significance level of 5%, was used to compare differences among groups.

## RESULTS

### Force system

The TLSs measured produced horizontal forces ranging from 116.7 gf to 498.9 gf (G1), -15.9 gf to 311.4 gf (G2), 35.8 gf to 452.6 gf (G3) and 121.9 gf to 463.7 gf (G4) between 0.5 and 5 mm of activation. TLSs from G2 produced the lowest initial forces of deactivation compared to the other three groups. (Table 2 and Fig 4) The TLSs from G3 showed the highest LD rates (93.7 gf/mm), followed by G1 (85.5 gf/mm), and by G2 and G4 (72.7 and 76.0 gf/mm, respectively), which showed similar LD rates. 

**Table 2:** - Means and standard deviations for forces (gf), neutral position (mm), LD ratio (gf.mm) and ANOVA results over a range of 5 mm of deactivation.

	**1**		**2**		**3**		**4**		***p* (95%)**				
	**Mean**	**SD**		**Mean**	**SD**		**Mean**	**SD**		**Mean**	**SD**		
5 mm	498.9	18.7	B	311.4	79.92	A	452.58	26.73	B	463.65	13.8	B	< 0.001
4.5 mm	453.74	18.78	B	273.67	72.85	A	406.47	24.43	B	424.35	13.91	B	< 0.001
4 mm	414.1	18.41	C	238.37	68.59	A	362.21	24.27	B	388.57	14.05	BC	< 0.001
3.5 mm	373.93	18.3	C	203.04	64.58	A	317.58	24.1	B	352.48	14.22	BC	< 0.001
3 mm	333.22	18.18	C	167.3	60.78	A	272.42	24.29	B	315.85	14.48	C	< 0.001
2.5 mm	291.66	18.16	C	131.48	57.3	A	226.65	24.4	B	278.65	14.92	C	< 0.001
2 mm	249.29	18.29	C	95.34	54.15	A	180.0	24.58	B	240.77	15.5	C	< 0.001
1.5 mm	206.05	18.51	C	58.55	51.22	A	132.61	24.82	B	201.93	15.94	C	< 0.001
1 mm	161.89	18.86	C	21.53	48.83	A	84.48	25.06	B	162.28	16.4	C	< 0.001
0.5 mm	116.66	19.42	C	-15.87	47.1	A	35.77	25.31	B	121.94	17.29	C	< 0.001
Neutral position	-0.86	0.22	A	0.72	0.62	C	0.13	0.29	B	-1.13	0.27	A	< 0.001
LD	85.5	3.04	B	72.7	12.51	A	93.71	2.59	C	75.96	3.01	A	< 0.001

Different letters indicate group differences.

**Figure 5:**
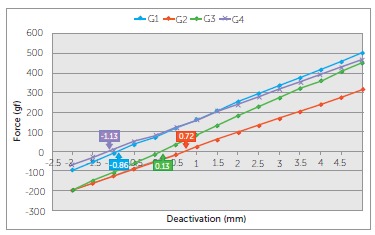
- Figure 4 slightly modified. The x- intercepts, pointed by the arrows, depict the amount of horizontal overlap of the vertical extensions of the TLSs in "neutral position" and were calculated by linear interpolation.

The amount of overlap of the vertical extensions of TLSs (in neutral position) was different between G2 (0.72 mm) and G3 (0.13 mm), which, on the other hand, were different from the similar overlap that occurred between G1 and G4 (-0.86 mm and -1.13 mm, respectively) (Table 2and Fig 5).

**Figure 6:**
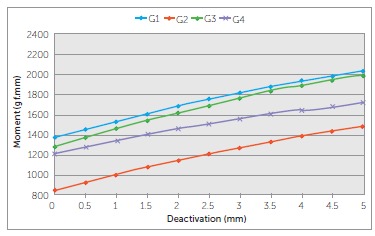
- Moments produced by the b-Ti TLSs over a range of 5 mm of deactivation.

TLSs delivered moments that ranged from 1452.0 gf.mm to 2030.7 gf.mm (G1), 919.0 gf.mm to 1482.0 gf.mm (G2), 1366.1 gf.mm to 1992.1 gf.mm (G3) and 1276.9 gf.mm to 1721.3 gf.mm (G4) between 0.5 and 5 mm of activation. G1 and G3 produced the highest levels of moments initially, while G4 produced lower moments than G1, but the values were similar to G3 and G2. G2 was different from all other groups. (Table 3 and Fig 6)

**Table 3:** - Means and standard deviations for moments (gf.mm) and ANOVA results over a range of 5 mm of deactivation.

	**1**		**2**		**3**		**4**		***p* (95%)**				
	**Mean**	**SD**		**Mean**	**SD**		**Mean**	**SD**		**Mean**	**SD**		
5 mm	2030.7	290.16	C	1482.0	278.8	A	1992.1	179.27	BC	1721.3	110.65	AB	< 0.001
4.5 mm	1977.8	282.51	C	1430.7	272.23	A	1941.5	177.57	BC	1677.7	113.48	AB	< 0.001
4 mm	1934.9	276.38	C	1382.3	269.75	A	1891.9	170.27	BC	1641.3	116.98	AB	< 0.001
3.5 mm	1874.7	265.35	C	1329.43	268.39	A	1837.9	169.29	BC	1601.4	117.46	B	< 0.001
3 mm	1812.1	253.31	C	1271.4	267.33	A	1761.0	148.82	BC	1557.1	119.2	B	< 0.001
2.5 mm	1745.3	236.17	B	1211.3	268.41	A	1687.6	125.73	B	1507.7	117.62	B	< 0.001
2 mm	1683.3	224.98	B	1149.1	269.11	A	1615.6	124.93	B	1455.7	114.46	B	< 0.001
1.5 mm	1597.2	180.45	B	1075.8	267.83	A	1538.2	126.97	B	1402.0	112.18	B	< 0.001
1 mm	1527.8	174.18	B	1000.2	265.97	A	1459.4	131.14	B	1339.2	106.87	B	< 0.001
0.5 mm	1452.0	169.17	B	919.0	265.91	A	1366.1	132.17	B	1276.9	106.35	B	< 0.001

Different letters indicate group differences.

**Figure 7:**
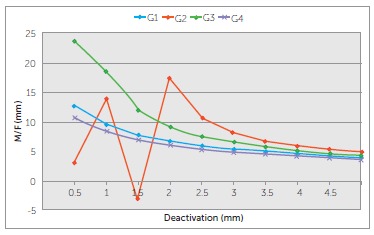
- MF ratio produced by the b-Ti TLSs over a range of 5 mm of deactivation.

G2 and G3 showed the highest MF ratios initially (4.9 and 4.4 mm, respectively), followed by G1 (4,1 mm) which was similar to G3 and G4 (3.7 mm). From 1.5 mm of deactivation on, there was no difference among groups. (Table 4 and Fig 7). 

**Table 4:** - Means and standard deviations for MF ratios (mm) and ANOVA results over a range of 5 mm of deactivation.

	**1**		**2**		**3**		**4**		**p (95%)**				
	**Mean**	**SD**		**Mean**	**SD**		**Mean**	**SD**		**Mean**	**SD**		
5 mm	4.07	0.55	AB	4.86	0.72	C	4.4	0.33	BC	3.72	0.28	A	< 0.001
4.5 mm	4.36	0.59	AB	5.35	0.86	C	4.78	0.37	BC	3.96	0.33	A	< 0.001
4 mm	4.67	0.64	AB	5.98	1.06	C	5.23	0.41	BC	4.23	0.37	A	< 0.001
3.5 mm	5.02	0.69	AB	6.83	1.43	C	5.8	0.49	B	4.55	0.42	A	< 0.001
3 mm	5.44	0.73	AB	8.12	2.16	C	6.48	0.5	B	4.94	0.49	A	< 0.001
2.5 mm	5.99	0.77	A	10.4	4.02	B	7.48	0.74	A	5.43	0.58	A	< 0.001
2 mm	6.77	0.89	A	17.25	13.64	B	9.09	1.15	A	6.08	0.7	A	0.003
1.5 mm	7.79	1.01		-3.03	49.35		11.91	2.18		6.99	0.9		0.537
1 mm	9.53	1.42		13.76	29.44		18.74	6.21		8.35	1.26		0.425
0.5 mm	12.71	2.35		3.09	71.76		23.65	131.14		10.71	2.1		0.927

Different letters indicate group differences.

### Wire dimensions

Significant differences were found among the sizes of wires (Table 1). Groups 1, 3 and 4 had the same height (0.0165-in) which was larger than the dimension of G2 (0.0161-in). Regarding the width of the wires, G1, G2 and G4 had the same dimensions (0.024-in; 0.024-in; 0.0242-in, respectively), while G3 (0.0244-in) was different from all of them, except for G4. 

### Mechanical properties

b-Ti wires from G3 showed the highest YM at 56.5 GPa, which was similar to G1 (51.0 GPa) and G2 (50.3 GPa). G4 showed the lowest YM (48.1 GPa), which was different from G3, but similar to G1 and G2 (Table 5).

**Table 5:** - Means and standard deviations for YM and ANOVA results over a range of 5 mm of deactivation.

**Group**	**Mean Young's test**				
	**Modulus (GPa)**	**SD**	**Range**	***p* (95%)**	
1	51.0	ab	1.7	3.9	0.012
2	50.3	ab	2.9	7.3	
3	56.5	b	6.2	14.6	
4	48.1	a	0.7	1.6	

Different letters indicate group differences.

### Chemical composition

The wires from all groups showed similar composition regarding Titanium (Ti), Molybdenum (Mo), Zirconium (Zn), Sulfur (S), Tin (Sn) and Phosphorus (P), except for the wires from G2 which had a higher concentration of Ti and a lower concentration of Mo (Table 6). 

**Table 6:** - Composition of b-Ti wires used in the study (in % of total composition).

**Group**	**Ti**	**Mo**	**Zr**	**S**	**Sn**	***p***	**Sum (%)**
1	68.4	13.6	6.1	4.7	3.4	3.2	99.4
2	72.6	11.3	6.2	4.0	2.9	2.5	99.6
3	68.1	13.0	5.5	5.8	4.0	3.6	100.0
4	69.7	13.3	5.9	4.2	4.0	2.8	100.0

## DISCUSSION

All groups produced similar force levels at 5 mm of deactivation, except for G2 which showed lower forces. Even though there were differences found in the dimensions of the wires, these differences were small (ranging from 0.0004 to 0.0002) and probably unable to influence the results significantly. The neutral position of the loops is probably the factor that can best explain these differences. The amount of overlap of the vertical extensions of the loops, when in neutral position, may create an over or underactivation of the loop initially, which is the consequence of the shape of pre-activation of the loop.^17^^,^ 18 ^,^ 21 In this study, however, the groups tested had the same pre-activation shapes, which does not explain the differences found in neutral position. Chemical differences among wires, on the other hand, could influence how each particular brand of b-Ti responds to the bends made to the design of the loop, and could, therefore, play a major role in causing these differences.^3^

All groups had similar composition, except for G2 which showed a higher percentage of Ti and a lower percentage of Mo. Even though this could explain only partially the differences found (G2 produced lower forces initially), it cannot explain why all groups, but G1 and G4, were different among themselves regarding the neutral position. The differences might finally be explained by other factors, such as the manufacturing process, which can alter the wires properties.^3^

Throughout deactivation, the TLSs from all groups acted differently, except for G1 and G4. This result can be substantiated by the differences found in LD rate. These differences can be explained by the design,^9^^-^ 13 method of pre-activation,^11^^,^ 12 ^,^ 13 ^,^ 16 ^-^ 18 and chemical composition of the wire^3^^,^ 19 ^,^ 20 and, finally, by the method of manufacture of the wires.^3^ In this study, the design, method of pre-activation and size of the wire were controlled. The similar chemical composition among groups, except for G2, could only partially explain the differences because it does not explain the different behavior of G3. The physical properties of the wires due to the manufacturing process might play a role on the subject, as well as how each beta-titanium wire brand responds to bends in the wire and stress relief, since the tensile test made found similar LD rates (Young's modulus) among all groups.^21^^,^ 22 ^,^ 24

**Figure 8:**
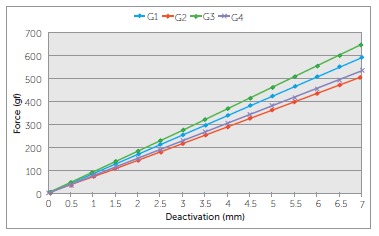
- Figure 4 mathematically adjusted in order to isolate the effect of the overactivation on the force produced by the groups caused by the overlapping of the vertical extensions.

**Figure 9:**
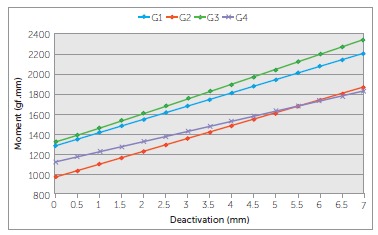
- Figure 6 mathematically adjusted in order to isolate the effect of the overactivation on the moments produced by the groups caused by the overlapping of the vertical extensions.

The differences found in the moments among groups were similar, as the ones found in the force levels. In this study, the differences among groups were probably due to neutral position and stress relief differences.^21^^,^ 22 ^,^ 24 This was expected, since the residual moment, or the moment produced by the concentrated bends, is related to the way each wire will behave to those particular bends.^11^^,^ 12 ^,^ 13 ^,^ 16 ^-^ 18 The effect of bends in the behavior of the wires can be confirmed if data are mathematically adjusted to neutral position, as already shown in the literature, because it can subtract the effect of how the wires respond to bends (Figs 8 and 9).^17^^,^ 18 This can be done by transposing the x-intercept of each line of the graph to the origin of the graph, along with every point of the line, isolating the effects of horizontal overlapping of the vertical extensions of the TLSs. It can be seen on the charts that the relation of force among groups is very similar to the relation of moments among them, if neutral position is not taken into consideration, and that TLSs behavior is pretty similar among groups.

**Figure 10:**
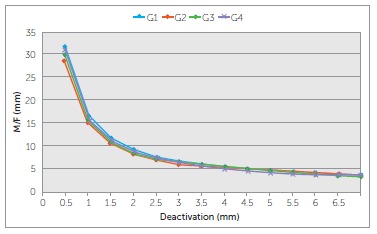
- Figure 7 mathematically adjusted in order to isolate the effect of the overactivation on the MF ratios produced by the groups caused by the overlapping of the vertical extensions The force systems developed by the groups was quite similar.

MF rate significantly varied among groups, since it is a proportion between the already variable force levels and moment levels. The fact that G1 and G4 produced similar MF rates is consistent with the similar behavior that they showed in neutral position (Table 2). G2 and G3 showed an inconsistent MF ratio on the last 2 mm of deactivation (G2) and very close to complete deactivation (G3) because of their positive neutral position (vertical extensions of the TLSs were apart). If the way each wire behaves in regards to neutral position was isolated (Fig 10) and removed experimentally, the MF ratios of all wires would be the same. Unfortunately, that is something that would not occur clinically. This, however, does not mean that the wires from G2 and G3 should not be used clinically. They can be used, but a different approach is needed when those wires are used in loops. If a TLS is to be used, the clinician should compensate the differences in the overlapping of the vertical extensions of the loop by opening less the inner "ears" of those two wire brands than what is normally recommended for G1 (TMA).^9^^,^ 22

## CONCLUSIONS

It can be concluded from the TLSs tested:

1. When they are made of different b-Ti wires, TLSs produce different forces, moments and LD ratios.
2. The cause of these differences is the way each wire behaves in relation to bends, thereby producing different shapes in neutral position.
3. Groups 1 (TMA) and 4 (CNA) showed a more consistent MF ratio throughout deactivation.
4. Even though groups 2 (Beta-Flexy) and 3 (Beta III TiMo) behaved differently from groups 1 and 4, this does not mean that they should not be used clinically, but a different approach is needed when loops are used.
